# The Pursuit of Happiness Measurement: A Psychometric Model Based on Psychophysiological Correlates

**DOI:** 10.1155/2014/139128

**Published:** 2014-04-30

**Authors:** Cipresso Pietro, Serino Silvia, Riva Giuseppe

**Affiliations:** ^1^Applied Technology for Neuro-Psychology Laboratory (IRCCS Istituto Auxologico Italiano), Via Pellizza da Volpedo 41, 20149 Milan, Italy; ^2^Department of Psychology, Catholic University of Milan, Largo Gemelli 1, 20123 Milan, Italy

## Abstract

Everyone is interested in the pursuit of happiness, but the real problem for the researchers is how to measure it. Our aim was to deeply investigate happiness measurement through biomedical signals, using psychophysiological methods to objectify the happiness experiences measurements. The classic valence-arousal model of affective states to study happiness has been extensively used in psychophysiology. However, really few studies considered a real combination of these two dimensions and no study further investigated multidimensional models. More, most studies focused mainly on self-report to measure happiness and a deeper psychophysiological investigation on the dimensions of such an experience is still missing. A multidimensional model of happiness is presented and both the dimensions and the measures extracted within each dimension are comprehensively explained. This multidimensional model aims at being a milestone for future systematic study on psychophysiology of happiness and affective states.

*It seems everyone has a view on happiness. Joan Collins, theDalai Lama and*

*over 100 others have released new titles on the subject since the beginning of 2001*

Richard Tooth

“The Psychology of Happiness (2nd Edition)”

Michael Argyle, Routledge

## 1. Introduction

“Life, Liberty and the pursuit of Happiness” is a sentence in the United States Declaration of Independence [1]. The sentence is considered an example of “unalienable rights” to be considered for all human beings.

Everyone is interested in the pursuit of happiness, but the real problem for the researchers is how to measure it. An interesting distinction is between Subjective Well Being (SWB), measures of happiness based on self-reports and surveys, and Objective Well Being, measures of observable variables, for example, based on life expectancy and other variables that we believe important for a good life. Among several methods between these two extremes, our aim is to deeply investigate happiness measurement through biomedical signals, using psychophysiological methods to objectifying the subjective experiences measurements.

Psychophysiology research has come to age to allow sophisticated and objective measurement of perceived experiences. However, there is still room for improvement in the research methods and in the consequent modeling of the involved processes.

The goal of our study was to model subjective experiences by measuring different dimensions of the affective states and the related psychological and physiological spheres.

According to the classic valence-arousal model [2, 3] for identifying affective states in subjects during an experimental session, we can consider the two dimensions of “activation,” namely, physiological arousal and emotional valence.

**Figure 1:**
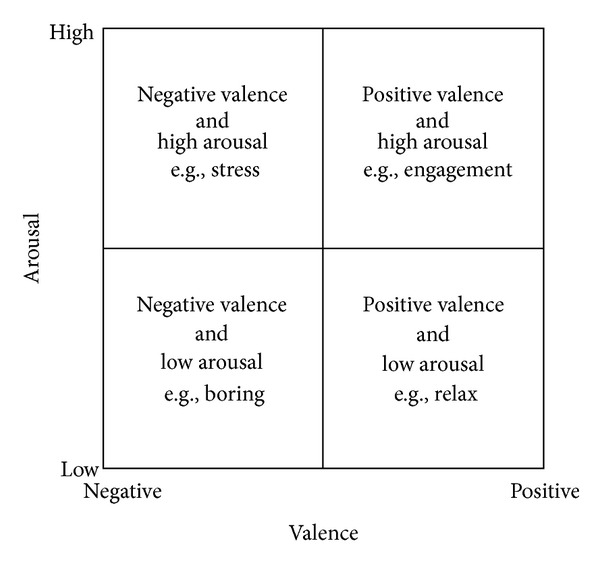
The classic valence-arousal model [2, 3] with the two dimensions of “activation”: physiological arousal and emotional valence.

Figure 1 offers an intuitive identification of affective states based on these two dimensions [3].

This approach has been extensively used in psychophysiological research as an objective way to measure affective states during a mediated experience [4–13]. More, recently an extensive research has been done also to discern different emotions by the means of cardiovascular measures [14, 15], and this hugely helps the analysis of affective states, by confirming the results that can be obtained, with specific patterns of the cardiovascular indexes [16–18].

Researchers measuring SWB have been able to deconstruct happiness into separate but related dimensions of positive effect, satisfaction, and negative effect [19–21]. Aroual-valence model can be used in this sense, but, however, we need to add another dimension: the life satisfaction.

Considering this new dimension, we add to the pursuit of proximal goals and immediate pleasure (hedonic enjoyment) also the long-term commitment to pursue “self-realization” (eudaimonia) [22, 23].

Life satisfaction can be considered as the opposite of depression. According to a classic study of Headey et al., “life satisfaction, is quite strongly (negatively) correlated with a distress dimension, depression; life satisfaction and depression are near opposites” [24]. Also recent studies keep the same relationship in clinical and experimental studies [25–36].

Psychophysiology of depression has been studied through Heart Rate Variability measurements historically [37–41], continuing also recently [42–53].

So psychophysiology of life satisfaction dimension can be easily computed referring to several studies and researches from the last twenty years.

**Figure 2:**
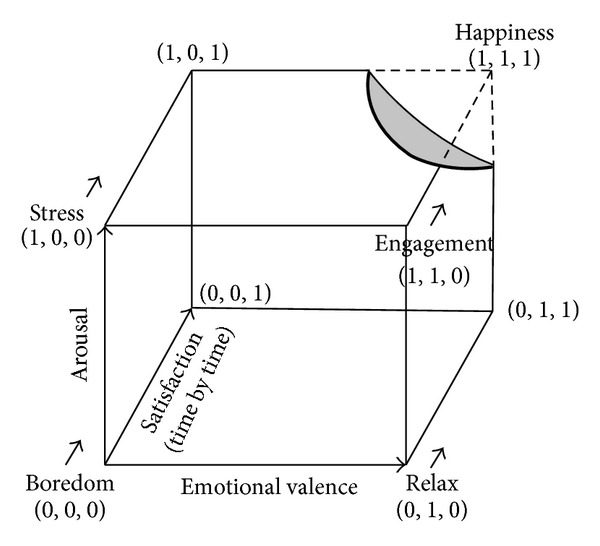
Arousal-Valence-Satisfaction space, identifying on frontal plan the valence-arousal model. The happiness is an extension of the engagement state with a higher level of life satisfaction.

In Figure 2, we represented the Arousal-Valence-Satisfaction space, identifying on frontal plan the valence-arousal model and, consequently, the relative affective states and the happiness as an extension of the engagement state due to a higher level of life satisfaction.

Thus, in the model, the happiness is identified as the situation in which subjects have a high physiological arousal, a positive emotional valence, and a high level of life satisfaction. Its vantage is to combine the three dimensions, making a specific experience measurable in a more effective way.

To summarize, we used psychophysiological measures to evaluate life satisfaction, emotional valence, and physiological arousal. In this perspective, engagement and happiness are strictly related to the link between short and long run: the more the subjects will be engaged and satisfied, the more they experience happiness, characterized by positive valence, high arousal, and high life satisfaction. Thus, we aimed to objectively model specific pattern of users' affective state in the Arousal-Valence-Satisfaction plane.

## 2. Model Hypotheses

The model purpose is to work on typical ground truths in a multidimensional space of objectively measurable variables and to explore if a possible subjective experience can be identified as a happiness experience. Thus, for an effective assessment of subjects' experience, we have to identify stable ground truths in the tridimensional space that we considered.

Several studies, recently, established stable ground truths. Some of the most important databases at this purpose are the IAPS (for the images) [54], the IADS (for the audios) [55], the Affective Norms for English Words (ANEW) [56], the Affective Norms for English Text (ANET) [57], and the Age-Dependent Evaluations of German Adjectives (AGE) [58]. In these databases, several stimuli are classified on the basis of physiological arousal and emotional valence and are used also to investigate other dimensions, like, for example, in the recent study of Leite and Colleagues [59]. These databases have been also investigated in hundreds of psychophysiological studies and also with patients [60], which made evidence of objectivity and effectiveness of the used stimuli.

These and other databases have a great role in psychophysiology, neuroscience, and many other related fields; however, an aspect that often is not considered is that to experiment beyond the basic research, in particular, into the applied field, hugely complexify the situations.

The pursuit of happiness measurement may be a complex issue, and also, in the bidimensional models used at the moment, no one conceptualized correct research methods to analyze the outcome of the involved arousal and valence dimensions yet. This lack leaded to tons of studies where the analysis of statistical differences are considered just “good enough” to publish bidimensional model based on arousal-valence plan, that however considered only single variables without going deeper on the combination or the relations among them.

One statistically correct study, that got beyond the statistical differences, is of Von Leupoldt and Colleagues [61] where an analysis of polynomial contrasts has been conducted to analyze the trends. Another relevant study has been really well conducted by Grühn and Scheibe [10], where the relations between arousal and valence are taken into consideration.

Of course, many studies considered sophisticated statistical technique to analyze the data results; however, no one considered yet combinations and analyses beyond the statistical differences, that come to be essential in multidimensional studies.

The pursuit of the happiness measurement regards a plenty of fields about the human sciences. Typical examples are the studies on ergonomics but also all the studies in the field of positive psychology, where the idea is to investigate the optimal experience and the flow state [62, 63]. Also a new emergent paradigm, the positive technology [64–66], seems going in the same direction.

Since this model aimed at being of a wide interest for several researchers, our approach will be toward the simplification of the complexity naturally embedded in a multidimensional model.

Also the statistical analyses are explained in details in a way to fit well also nonmathematical users, more descriptive than equation-based, however scientifically rigorous.

Based on this approach, we formulated three simple pseudohypotheses (following hypotheses).

Each hypothesis is based on one dimension of the multidimensional space in particular, we considered physiological arousal, emotional valence, and life satisfaction.

As ground truth, we defined two basic affective states, namely, “Relax” and “Stress,” to be elicited in a way that can represent the ground truth of relax state and stress state, respectively, in the tridimensional space considered. The way to elicit these two states strongly depends on the study that the researcher is carrying out. Relax can also be induced using panorama slides show with a soft music and a cognitive stress is easily induced by standard cognitive tasks, such as Stroop task or arithmetic task [17, 18, 67, 68].

Thus, in a possible experiment, it will be necessary to foresee at least three epochs: (1) the phenomenon that is investigated to be a happiness experience or not (following, to simplify, the happiness), (2) a relax epoch, and (3) a stress epoch.

This operation is to compare along each axis the happiness with a standard affective state elicited in the subject.

Each hypothesis needs also to be verified for the significant quadratic trend using the within-subjects contrasts (further specifications are given following, in a specific section).

The first hypothesis is on the dimension of physiological arousal. In particular, we hypothesize that a happiness experience leads to be more “activated,” that is, with an arousal activation similar to the stress states and enough different from the relax one.

The second hypothesis is on emotional valence, for which we expect, by definition, that a happiness experience is able to generate positive emotions and thus we hypothesize that emotional valence during a happiness experience is similar to a relax state and quite different from the stress state, that generates negative emotions.

The third hypothesis is on life satisfaction, for which we expect to have a high level of satisfying experience repeated time by time to make a happiness experience attracting continuously. The process is dissimilar to the one activated during a stress state, where the alertness toward the complex task leads users to move far from satisfaction. Thus we hypothesize that satisfaction during a happiness experience is different from both relax and stress states.

Thus, happiness experience differs from stress for the emotional valence and the satisfaction, being similar in the physiological arousal. This could be a great weakness of the model, since a few errors in measuring a variable could lead to opposite conclusions, considering a stressful experience as a happiness one. To avoid these misleading consequences is our strong suggestion to avoid considering the physiological arousal to measure happiness: this would bring to great errors.

A synthesis of the hypotheses is reported in Table 1. Of course, these hypotheses make it difficult to find a happiness experience; however, this is due to the fact that it is a complex phenomenon and not to the experimental variables, that are only used to objectively measure the subjects' states.

## 3. Psychophysiological Assessment

The multidimensional model aimed at measuring in an objective way the subjective experience. At this purpose we describe which biosensors and biomedical signals came to age to be considered consolidated enough to allow an objective measurement. More, it is to be taken into account that psychophysiological analysis is not easy and requires specific mathematical competences and not only sophisticated instruments; thus, following, we will give a short insight on the correct signal processing procedures necessary to extract the indexes (measures) that eventually can be used for the statistical data analysis.

### 3.1. Biosensors and Biomedical Signals

**Figure 3:**
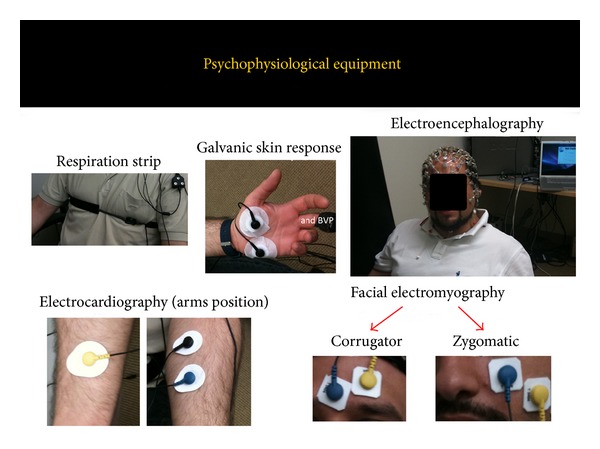
Psychophysiological equipment.

A number of biosensors and biomedical signals can be used; most biosensors are nowadays also wearable and their obtrusiveness is more and more reduced. Eye tracker acts at distance (about one meter); the other biosensors are electrodes-based, reading the electrophysiological signals by contact (see an example in Figure 3). The setting phase is simple but needs to be made by an expert researcher or physician to detect the exact locations or the signals extracted risk to be compromised.

Following a (nonexhaustive) list of typical biosensors/biomedical signals: electroencephalogram (EEG), galvanic skin response (GSR), electrocardiogram (ECG), blood volume pulse (BVP), respiration signal (RSP), eye tracker (ET), and facial electromyography (fEMG). In the next session a deeper insight on the biosensors use, and the sense of the extracted measures based on the multidimensional model will be given.

### 3.2. Signal Processing and Extracted Measures

Cardiovascular and respiratory activity is monitored to evaluate both voluntary and autonomic effect of respiration on heart rate, analyzing R-R interval extracted from electrocardiogram (ECG) and respiration (RSP) from chest strip sensor and their interaction. It is also possible to extract IBI (interbeat-interval) from blood volume pulse (BVP), that is an acceptable (even if worse) alternative to ECG's R-R. According to the guidelines of Task Force of the European Society of Cardiology and the North American Society of Pacing and Electrophysiology, typical heart rate variability (HRV) spectral indexes can be extracted to evaluate the autonomic nervous system response [16, 17, 69]. Spectral analysis can be performed using Fourier spectral methods. The rhythms can be classified as very low frequency (VLF, i.e., less than 0.04 Hz), low frequency (LF, from 0.04 to 0.15 Hz), and high frequency (HF, from 0.15 to 0.5 Hz) oscillations. This procedure allows us to calculate the LF/HF ratio, also known as the sympathovagal balance index. Cardiovascular and respiratory activity interaction can also be taken into account through Respiratory Sinus Arrhythmia (RSA) index [17, 69]. As temporal domain measures of heart rate variability are generally calculated NN50 index, that is, the number of interval differences of successive NN intervals greater than 50 milliseconds. This index describes the short-term NN variability. Just to simplify, NN intervals can be seen as a sort of beat-to-beat representation of heart rate; according to Camm and Colleagues [69], “In a continuous ECG record, each QRS complex is detected, and the so-called normal-to-normal (NN) intervals (that is, all intervals between adjacent QRS complexes resulting from sinus node depolarization) or the instantaneous heart rate is determined.”

Skin conductance mean (SC) can be extracted from a GSR biosensor. It is critical to remove possible movement artifacts before computing the index (since on the hand, it can be affected by consistent involuntary grasping). SC is an interesting measure, since the sweat glands are regulated by the sympathetic nervous system without a direct “contamination” of parasympathetic nervous system (that for example exists for HR). Thus SC is an excellent candidate to measure pure physiological arousal [70, 71].

The raw electromyography (EMG raw) is a collection of positive and negative electrical signals; their frequency and amplitude give us information on the contraction or rest state of the muscle. Amplitude is measured in *μ*V (microvolts). As the subject contracts the muscle, the number and amplitude of the lines increase; as the muscle relaxes, amplitude decreases [72–74]. It is generally considered the Root Mean Square (RMS) for rectifying the raw signal and converting it to an amplitude envelope [18, 75]. In particular cases we can also be interested in frequency, related to muscle fatigue [73]. There are a number of measures that can be extracted from this signal that depend on the muscle corresponding to the electrodes locations. For the model, there are three facial locations that give relevant information about emotional valence. In particular, RMS of EMG signal was recorded in correspondence of facial zygomatic major muscle (following EMG Zygomatic), that increases when positive emotions arise [72, 75]. On the other hand, the RMS of EMG signal recorded in correspondence was with facial corrugator supercilii muscle (following EMG Corrugator), that increases when negative emotions arise [72, 75]. Eventually, the RMS of EMG signal was recorded in correspondence of facial orbicularis oculi muscle underneath the eye with miniature electrodes muscle (following Startle Reflex), that is inversely proportional to the pleasantness of the stimuli [75].

Respiration signal can be elaborated to compute the respiration depth (RSP depth), the point of maximum inspiration minus the point of maximum expiration to be determined from the respiratory tracing. Smaller values indicate more shallow respiration and higher activation [18, 76]. It is also possible to calculate respiration rate (also measured in breaths per minutes) from peak-to-peak computing.

EEG signals need to be extensively worked to remove ocular artifacts and blinks, if possible basing on electrooculography (EOG) signals using automatic algorithm and subsequent visual inspection. Then the corrected matrixes can be computed to calculate means of the Beta EEG (e.g., 13–30 Hz) bands, of the Alpha EEG (e.g., 7–13 Hz) bands, and of the Slow Alpha EEG (e.g., 7–10 Hz) bands, one per each channel recorded, through spectral analyses [77–79]. Frontal EEG activation asymmetry has been generally used, giving evidences that greater left frontal activity seems to be higher related to positive emotional valence, whereas greater right frontal activity seems to be more involved in negative emotional valence [80]. Alpha index seems to be the most adapt to study the frontal EEG activation asymmetry [80]. Alpha Asymmetry index can be calculated in many different ways to take into account one hemispheric prevalence on the other one and correcting the sign accordingly. In calculating this index, it is crucial to consider that higher cortical activation is revealed by lower Alpha waves, and thus this needs to be considered in the computation and formula derivation. This Alpha Asymmetry is also a recognized index of depression [81–91] and hence can be used to measure life satisfaction and emotional valence.

Beta EEG bands (following Beta indexes) are often used to identify physiological arousal [79].

Using eye-tracker data, we can calculate the measure of cognitive and visual information processing, although they are limited in what they reveal about higher-order processes [92]. Eye movement data consist of moment-to-moment measures of the eyes' displacements along the vertical and horizontal axes (in mm) within the spatial working area of the monitor screen. The pupil size and gazes are acquired, based on the corneal reflection on the frontal surface of participants' eyes (caused by an infrared light source). After the experiment, the signals can be extracted and processed taking into account the blinks. The mean of pupil size (following pupil size) is considered as an important indicator of emotional arousal, that is, an arousal due to emotional stimuli, that is, one of the few indexes that take into account the physiological arousal as emotional consequences.

Every channel needs to be synchronously acquired at 2048 Hz and exported at last at 256 Hz sampling rate (256 records per second, one every 3.90625 millisecond). Some signals may be required to be extracted to a higher sampling rate (for example a minimum of 1024 is suggested for EMG signals).

To make interpretation relevant to actual users' affective state and to avoid contaminations, light and temperature sensors should be used to monitor the conditions of the room and, if possible, two three-axis accelerometers should be integrated into the biosensors and used to monitor subjects' stability and remove possible artifacts.

### 3.3. Synchronization and Epochs' Definition

Even if the use of eye tracker in the model is totally justified from pupil and gazes analyses are allowed by this tool, it is also crucial to underline its usefulness for synchronizing the psychophysiological signals within the experimental epochs. In fact, such synchronization become important when it is critical the timing for the presentation of the stimuli. In these cases, it become crucial to synchronize psychophysiological signals with eye-tracker data and to synchronize all these data with the sequences of stimuli presented to the subjects.

Usually, one way to overcome this problem is represented by the use of a webcam to record the stimuli screen or through a video screen capture program. However, these methods are not precise. In fact, even if the stimuli are synchronized with a computer clock, they require the visualization of a video to establish the periods, but this affects the time acquisition due to normal video latency. It is better, using the eye-tracker data extraction, to obtaine for each participant a matrix of gaze and pupil data corresponding to stimuli presentation, in particular, to collect a number of rows for each second (depending on the sampling rate used), thereby making it possible to establish the exact periods previously indicated.

A second step is represented by the synchronization of the stimuli with psychophysiological signals. In this case, it can use algorithms to synchronize eye-tracker systems with a psychophysiological device by using a photodiode, which can also be configured through a physical channel on the equipment used, just capturing the light (i.e., by identifying black and white). Practically, thanks to this photodiode actually applied on the screen and an algorithm [93]. Moreover, based on gazes and pupil signals acquired, it is possible to identify eye blinks, which enable us to align the matrixes containing the eye-blink data from gazes and pupil signals, with the matrixes containing the psychophysiological signals. Thanks to these procedures, it is possible to synchronize all signals and to correctly identify the experimental epoch, with an error of ±0.01 second [94].

To synchronize the presentation of objects with electrophysiological recordings, an interesting tool to keep in consideration is the box for interaction with objects (BIO) [95].

### 3.4. Personal Data Archiving

All participants data need to be memorized in encrypted and password protected files, possibly following the criteria to protect personal health information [96] and using PsychoPass or improved methods [97, 98] to generate and share passwords information among pairs.

## 4. Data Analysis

The model produces three successive observations of the same variable (measure considered for the analysis) on each subject. Repeated measures are defined as measurements sequentially conducted in time (temporal factor) or location (spatial factor) on the same subject. Repeated measurements are commonly employed to estimate measure parameters, investigate the factor effect on the process, and model and monitor the production and its process [99].

It is highly suggested to follow the recommendation of Bakker and Wicherts in reporting statistical results [100].

### 4.1. Repeated Measure Analysis of Variance

Repeated measure analysis of variance (rmANOVA, also known as ANOVARM) design requires three basic assumptions: (1) normal distribution of measures, (2) independent samples (if a between variable is taken into account, for example: depressed subjects versus nondepressed subjects), (3) homoscedasticity (equal variances of measures).

Additional assumptions are needed for rmANOVA as a result of the presence of correlations between measurements taken on the same subject at different levels (time, space, order, etc.).

In particular, an additional assumption to make the *F*-test of the repeated-measures valid is the sphericity (assumption of compound symmetry, that is, circularity of variance-covariance matrix) requiring homogeneity of the covariances among repeated measures.

A significant value for Mauchly's test of sphericity at *P* level .05 indicates that the assumption of homogeneity of covariance has been violated for some measures.

Commonly, two correction methods are used for adjusting to the *F*-test in terms of degree of freedom: the Huynh-Feldt and the Greenhouse-Geisser test [101, 102]. In these cases, corrected *P* values need to be reported accordingly.

Girden [103] recommended that if epsilon (Greenhouse-Geisser estimate) is larger than 0.75, then the correction according to Huynh and Feldt should be used. On the other hand, if epsilon is smaller than 0.75, then the more conservative correction according to Greenhouse-Geisser is preferred.

### 4.2. Pairwise Comparisons

A common error on data analysis is the use of paired-samples *t*-test to compare couples of repeated measures. In the model, it is necessary to have a precise idea of each dimension, by comparing “relax versus happiness” and “happiness versus stress,” through the use of pairwise comparison adjusting the alpha level to avoid an inflated type I error rate making multiple statistical comparisons (using, for example, Bonferroni correction). Most statistical softwares used for behavioral sciences have an embedded tool to correct these values.

It is also possible to use contrasts, based on *F* test, for comparison. In particular, simple contrasts (baseline versus each other level) or repeated contrasts (comparison of adjacent levels) can be used.

### 4.3. Polynomial Contrasts

Polynomial a priori contrasts can be computed by testing the hypothesized quadratic trends for main effects in physiological arousal (hypothesis 1) measures, with higher values for the happiness and stress epochs in comparison with the relax epoch. Polynomial a priori contrasts can also be performed by testing the hypothesized quadratic trends for main effects in emotional valence (hypothesis 2) and life satisfaction (hypothesis 3) measures, with positive values for the relax and happiness epochs in comparison with the stress epoch.

A monotonic trend with measures increasing (hypothesis 1) or decreasing (Hypotheses 2 and 3) from the relax to happiness to stress epochs is also expected.

### 4.4. Sample Size, Sensitivity, and Post Hoc Power Analysis

Hypothesis test tells us the probability of a result of that magnitude occurring, if the null hypothesis is correct (i.e., there is no effect in the population). It does not tell us the probability of that result, if the null hypothesis is false (i.e., there actually is an effect in the population).

Specifically, we consider the effect size, the sample size, and the criterion required for significance (*α*, where *α* is probability of type I error). These three factors, together with power (1 – *β*, where *β* is probability of type II error), form a closed system; once any three are established, then the fourth is completely determined [104].

**Figure 4:**
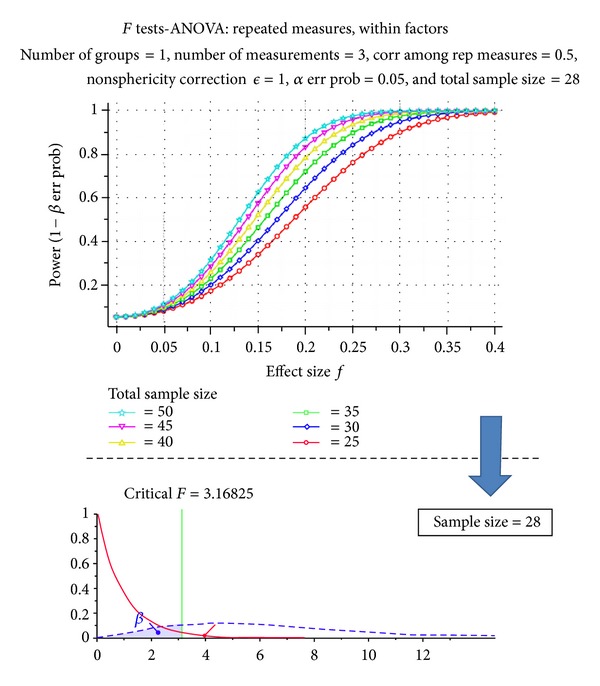
Lower effect sizes lead to a necessary increase of sample size to achieve the same minimum power. Estimated sample size from a literature analysis is of at least 28 participants.

A sample size calculation for the model experimental design, based on rmANOVAs, leaded an estimation of minimum 28 subjects to be used for possible experiments, in order to achieve a minimum power of 0.8, considering a medium effect size of 0.25 and a significance level of 0.05, and sphericity assumption satisfied (see Table 2) [104]. As can also be seen from Figure 4, a lower effect size leads to a necessary increase of sample size to achieve the same minimum power.

Once the results are computed, a power analysis can be used to anticipate the likelihood that the study yielded significant effects. In particular, the goal of a post hoc power analysis is to compute achieved power, given the effective other three factors, which can be read or deducted by data (output of statistical data analysis). Since many statistical softwares give *pη* ^2^ (partial eta-square) values instead of cohen's *f* effect size, it is important to compute *f* = sqrt[*η* ^2^/(1 − *η* ^2^)], where sqrt is for square root calculation.

According to post hoc power analysis, some significance level could be high informative even if slightly higher than .05 (it depends on achieved *pη* ^2^ for that measure).

## 5. Hypotheses Testing

As explained and reported in Table 1, three hypotheses are given for the model. Each hypothesis refers to one dimension of the model. A detail on expected measures within each hypothesis/dimension is presented, and comparisons with the ground truths are discussed. Consideration on statistical significance and polynomial contrasts is also taken into account.  Table 3 summarizes the measures within each hypothesis.

### 5.1. Hypothesis 1: Physiological Arousal

HR, SC, Beta indexes, and pupil size should be lower in relax epoch and higher in stress epoch; respiration depth for physiological arousal is in the smaller-is-higher form; consequently it is expected to be higher in relax epoch and lower in stress epoch, because relax produces lower physiological arousal and stress produces higher physiological arousal. Values of indexes for physiological arousal during happiness should be more similar to the ones in stress epoch. Practically, in both stress and happiness, the subject is in a situation of elevated physiological activation. Thinking of positive and engaging situation, such as gaming or other highly involving situations, it is easy to understand this state of higher activation in happiness.

Repeated measures ANOVAs can be used with epoch type (relax, happiness, and stress) as the within-subject variable for all the indexes used to measure physiological arousal. A main statistical significant effect of epoch type is expected for all the measures. Pairwise comparisons using the Bonferroni (or others) corrections can be used to reveal if there are statistically significant differences between relax epoch and happiness for all the indexes and no statistically significant differences between happiness and stress epoch for all the indexes (i.e., these two epochs are supposed to be so similar to produce no differences in arousal activation).

Polynomial a priori contrasts resulting by testing the hypothesized quadratic trends for main effects, with lower value of respiration depth and higher values for all the other indexes, can be used to measure physiological arousal for the happiness and stress epoch in comparison with the Relax epoch.

### 5.2. Hypothesis 2: Emotional Valence

EMG Zygomatic and Alpha Asymmetry measures should be higher in relax epoch and lower in stress epoch; EMG Corrugator and Startle Reflex measures for emotional valence are in the smaller-is-higher form; consequently, they are expected to be lower in relax epoch and higher in stress epoch, because relax elicits positive emotional valence and stress elicits negative emotional valence.

Values of indexes for emotional valence during happiness should be more similar to the ones in relax epoch. Practically, in both relax and happiness experience, the subjects are in a situation of positive emotional valence, that is, pleasantness. Being in relaxing situations or being in happiness experience leads to have positive emotional valence.

Repeated measures ANOVAs can be used with epoch type (relax, happiness, and stress) as the within-subject variable for all the indexes was used to measure emotional valence. A main statistically significant effect of epoch type is expected for all the measures. Pairwise comparisons using the Bonferroni (or others) corrections can be used to reveal if there are statistically significant differences between happiness and stress epoch for all the indexes and no statistically significant differences between Relax epoch and Happiness for all the indexes (i.e., these two epochs are supposed to be so similar to produce no differences in emotional valence).

Polynomial a priori contrasts resulting by testing the hypothesized quadratic trends for main effects, with higher values of EMG zygomatic and Alpha Asymmetry indexes and lower values for EMG Corrugator and Startle Reflex indexes can be used to measure emotional valence for the relax epoch and happiness in comparison with the stress epoch.

### 5.3. Hypothesis 3: Life Satisfaction

LF/HF and LF power measures are supposed to be lower in relax epoch and higher in stress epoch; NN50, RMSSD, and HF power measures for depression are in the smaller-is-higher form; consequently, they are expected to be higher in relax epochs and lower in stress epoch, because relax produces lower anxiety and stress produces higher anxiety. Since depression is negatively related to life satisfaction, the above indexes will be exactly the opposite to measure higher happiness.

Happiness is characterized by a low level of depression, resulting having a high level of life satisfaction, that is, different from the relax and stress states.

Repeated measures ANOVAs can be used with epoch type (relax, happiness, and stress) as the within-subject variable for all the indexes was used to measure depression. A main statistically significant effect of epoch type is expected for all the measures. Pairwise comparisons using the Bonferroni (or others) corrections can be used to reveal if there are statistically significant differences between happiness and both relax and stress epochs for all the indexes.

Polynomial a priori contrasts resulting by testing the hypothesized quadratic trends for main effects, with higher values of NN50, RMSSD, and HF power indexes and lower values of LF/HF and LF power, can be used to measure lower life satisfaction for the relax and stress epochs compared to happiness.

### 5.4. Linear-Quadratic Trend Coexistence

A linear monotonic significant trend with measures changing from the relax to happiness to Stress epochs may also result for all the indexes considered in physiological arousal and emotional valence but not in life satisfaction (where only a quadratic form is expected, being the two extremes—relax and stress—at the same low level and the happiness to a higher level, by definition).

There are three possible situations of statistically significant polynomial contrasts:

1. only linear trend is statistically significant;
2. both linear and quadratic trends are statistically significant;
3. only quadratic trend is statistically significant.

**Figure 5:**
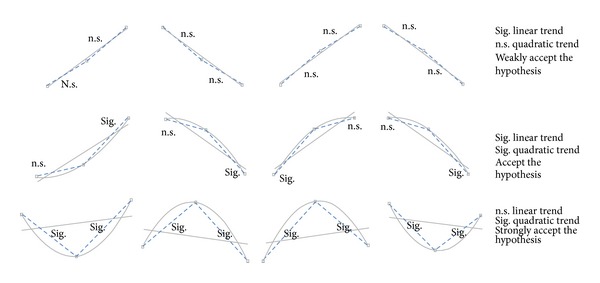
Schema of the listed situations and graphical representations of the linear-quadratic trend coexistence. Here, we illustrate linear or quadratic relationships among the three states regardless of Cartesian representation.

Figure 5 reports a schema of the listed situations and graphical representations of the linear-quadratic trend coexistence.

The first situation, with only statistically significant linear trend, in the model denotes a situation, where the happiness is in the middle between relax and stress epochs, but is not clear if it is closer to one or the other. Generally such a situation comparing the happiness with the other two states (relax and stress) leads to statistically significant differences in both the directions and becomes even more difficult to make a decision. In these cases, it becomes relevant to have more than one measure for the considered dimension, in order to strengthen the possible acceptance or rejection of the hypothesis.

In the second situation, both linear and quadratic trends are statistically significant. This denotes that there are two consecutive states with similar values, in our model “relax and happiness” or “happiness and stress.” In this situation comparing the happiness with the other two states (relax and stress), it is probable to have statistically significant differences in only one direction, which would make it more easier to understand the happiness state. In this case, linear and quadratic trends and statistical significances provide more information about the closeness of the happiness to each conditions (“closer to relax” or “closer to stress”), strengthening the decision.

In the third situation listed before, an elevated increase in quadratic trend may lead to the loss of the monotonic trend and no statistical significant linear trend. In the model when this happens leads to strength the hypothesis. For example, let us consider the following scenario situation. Emotional valence measured through the EMG zygomatic shows (1) a significant quadratic trend and no significantly linear trend; (2) from simple (or repeated) contrasts (*F*-test based) or from the pairwise comparison (*t*-test based, with correction) result, statistically significant differences between happiness and stress and nonstatistically significant differences between relax and happiness, that is correct being EMG Zygomatic and index of emotional valence; (3) from descriptive results that the values for relax and happiness are higher than the values in stress, if not it means that EMG zygomatic is behaving like an EMG Corrugator and a deeper intelligence on signal processing or channels naming would be very suggested.

In the scenario situation just described the EMG Zygomatic is so high during happiness experience to generate a strong quadratic trend. This let us suppose, practically, that emotional valence has been more positive during the happiness experience, that actually strengthen our hypothesis.

**Figure 6:**
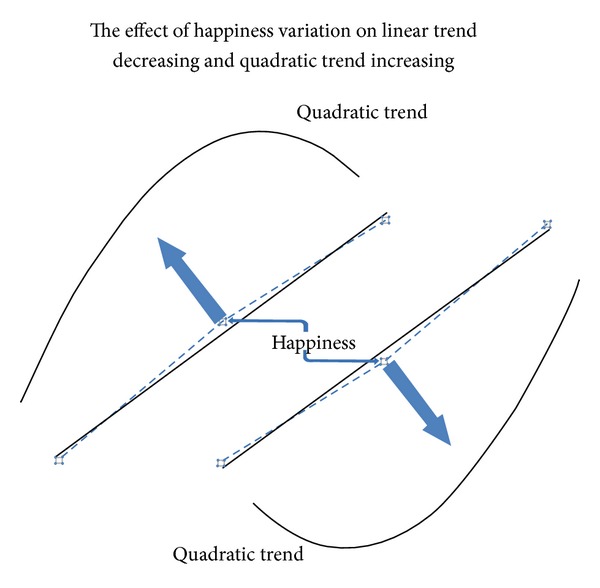
Dynamics of a happiness state,* ceteris paribus*. Here we illustrate linear or quadratic relationships among the three states regardless of Cartesian representation.

Figure 6 shows the dynamics of a happiness state,* ceteris paribus*. While the happiness moves toward a new state, the quadratic trend increases (and its significance level decreases) and the linear trend decreases (and its significance level increases).

### 5.5. Other Elements against or Supporting the Hypotheses

In the multidimensional model, it is necessary to evaluate the strength or weakness of each hypothesis, working on the several dimensions step by step, one dimension per time.

Within each hypothesis, the possible weakness of a measure needs to be considered in a wider context. In the case of unexpected values of a measures beyond the typical considerations that arise from the intrinsic imperfection of statistics (remembering the tails of a normal distribution), it is crucial to consider the effect that a dimension may have on the measure of another dimension. Typical example is the HR index that, even if it is recognized as a physiological arousal measure, may show unexpected values due to the effect of the baroreceptor reflex that causes heart rate to decrease also producing a variation in sympathovagal balance and in particular in a part of the LF, known as Mayer waves, at 0.1 Hz [105]. This means that there is a complex interaction between physiological arousal and depression that may produce adjustment to a part of HR index. This becomes a great limitation if complex interaction between variables are not checked by the means of correct indexes in all the dimension. In the example of HR, a lower than expected HR needs to be inspected using the several cardiovascular measures available, even if calculated for another dimension (depression and life satisfaction, in this case).

## 6. Discussion

We presented a multidimensional model to measure happiness by the means of psychophysiological correlates.

**Figure 7:**
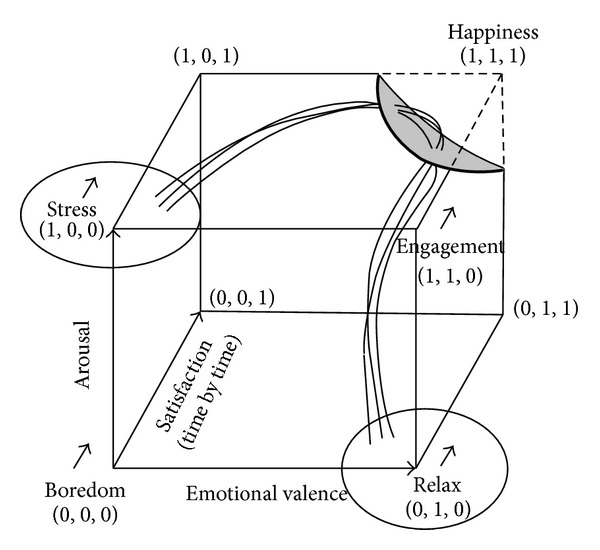
A representation of possible paths from relax to happiness experience to stress states.

Dimension considered was physiological arousal, emotional valence, and life satisfaction. Psychophysiological measures can be an extremely useful source of knowledge in each domain considered. A correct and complete statistical analysis based on rmANOVAs and polynomial contrasts can be used to create a map of the affective states and detect in a rigorous empirical way if happiness arose.  Figure 7 is a representation of possible paths from relax to happiness experience to stress states.

Future works should focus on a deeper classification of the dimensions and their relationships; in fact, it is crucial to investigate measures in a dimension and also in a wider view, considering the effect of a dimension on another dimension.

The multidimensional model is based on a statistical approach. Practically three sessions of physiological data are collected: during a relax epoch, during a stress epoch, and during the condition to verify, supposed to be during a happiness experience.

The disadvantage of such an approach is that it is necessary to have well-ested ground truths to which the experimental condition can be compared. However we saw that many databases with well-classified affective states are available and use our same classifications, among the others (IAPS, IADS, etc.).

Another limit of this approach is that the results, to be robust, need a large sample, of minimum 28 participants, but better if they are more than 35 since the effect size could be less than 0.25 once actually calculated. This is a practically a huge problem; in fact doing an experiment with psychophysiological signals is really demanding, requiring big efforts and attention during the recording phase and huge works and abilities* a posteriori*. In fact, the signal processing phase may also take long time, for at least two reasons: (1) the huge amount of data, just to give an idea 5 minutes of recording sampled at 256 Hz (it is the minimum, but some signals require also 1024 or more), means a matrix with a minimum of 76,800 rows and one column per each channel recorded, that then need to be preprocessed, filtered, and computed for a certain number of indexes extracted; (2) most signals need to be visually inspected for a corrected signal processing procedure; for example, from ECG signals it is necessary to extract R-R waves to compute cardiovascular indexes that we described; however, a good automatic detection algorithm may detect correctly 95% of R-peaks, and this means that the researcher needs to look at all the signals and correct the problems accordingly. Due to these considerations and to the general complexity of psychophysiological experiments, to collect data from 28 participants or more is not simple at all and often requires a well-consolidated team devoted to it.

On the other hand the advantage of such a complex approach is that when the experiments are conducted in a rigorous way, the results describe the subject reactions objectively allowing the researchers to achieve meaningful conclusions, of course in the limit of the study.

More, the increased computational capacity and the huge advancement in the field of artificial intelligence made another approach available that also gained a good credibility, that is, the affective computing. According to Rosalind Picard, who coined the term, “affective computing is computing that relates to, arises from, or deliberately influences emotion or other affective phenomena.” [106].

But how affective computing is related to our pursuit of happiness? Which advantages may offer? To answer these questions we need to determine to which extent the affective computing approach is different from the statistical one.

**Figure 8:**
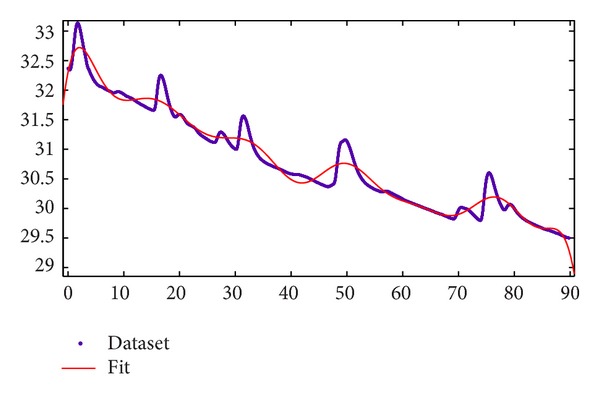
An SC signal processed in real time with a Fit function, based on a sum of sinusoids (*f*(*x*) = *a*1∗sin⁡(*b*1∗*x* + *c*1) + *a*2∗sin⁡(*b*2∗*x* + *c*2) + *a*3∗sin⁡(*b*3∗*x* + *c*3) + *a*4∗sin⁡(*b*4∗*x* + *c*4) + *a*5∗sin⁡(*b*5∗*x* + *c*5) + *a*6∗sin⁡(*b*6∗*x* + *c*6) + *a*7∗sin⁡(*b*7∗*x* + *c*7) + *a*8∗sin(*b*8∗*x* + *c*8).). Horizontal axis represents time (in seconds) and vertical axis represents the SC level (in microsiemens (µS)).

Let us imagine that we want to build an mp3 reader that automatically plays Mozart when you are stressed, recognizing if you become happy. Is that possible? This is a typical research question that arises from affective computing approach. The main difference from a statistical approach is that the target in this case is the real-time monitoring of a single subject's affective states (e.g., in Figure 8 is represented an SC signal processed in real time and the computed Fit function, based on a sum of sinusoids model (*f*(*x*) = *a*1∗sin⁡(*b*1∗*x* + *c*1) + *a*2∗sin⁡(*b*2∗*x* + *c*2) + *a*3∗sin⁡(*b*3∗*x* + *c*3) + *a*4∗sin⁡(*b*4∗*x* + *c*4) + *a*5∗sin⁡(*b*5∗*x* + *c*5) + *a*6∗sin⁡(*b*6∗*x* + *c*6) + *a*7∗sin⁡(*b*7∗*x* + *c*7) + *a*8∗sin(*b*8∗*x* + *c*8).)). In this case the signal analysis can be an automatic and continuous process that collects, seconds-by-seconds, physiological signals, classifies them, and gives an immediate output of the pattern recognition to the subject; if not correct the system may be instructed to autocorrect and improve its recognition algorithms, by the means of data mining techniques.

Of course affective computing is a fascinating field; however, it contains intrinsic limitations, mainly due to lacking in classification. It is in fact complex to recognize an affective state after months of signal processing and data analysis, let imagine in real time. However the big advantage of affective computing is that it fostered the development of a plenty of classification methods with a vivid international discussion at really high scientific levels. The continuous development of new artificial intelligence techniques further enriches this scenario.

At this very point, thus, the real question is if it can be possible to integrate the two approaches for a better measurement of the experience and a better detection of happiness.

A possible approach could follow these steps: (1) an experiment with a statistical approach, to create a model of the experience based on random sampling; (2) data reduction and extraction, that is, on the basis of the results obtained with the experiments to find the most informative measures for that specific happiness experience; and (3) experience tracking and automatic detection of happiness experience, based on the specific measures extracted. Once the measures are extracted this can constitute a new sample for statistical analysis for the perfection of the process.

We created in this way a closed loop of analysis aimed at fostering a better detection and measurement of happiness experiences.

However, also this approach presents some limitations; in fact while the first phase is a standard experiment in a laboratory setting, the last phase requires a naturalistic setting to be really effective, at least for many kinds of tracked experiences.

Naturalistic setting means considering “real mobile” setting, that is,* in vivo* experiments out of the lab, using a computerized ecological momentary assessment [107–109], with the combined integration of wearable biosensors.

The last challenge that we need to consider for the multidimensional model presented is the possible use of other measures collected by the means of less obtrusive biosensors, however being objective and reliable. Unobtrusiveness in data collection will probably represent the next most exciting challenge in psychophysiology.

## Figures and Tables

**Table 1:** Our pseudohypotheses (~ is for similar, > is for greater than, and < is for lower than).

Hypothesis	Dimension	Comparing happiness with	
		Relax	Stress
Hp1	High physiological arousal	Happiness > relax	Happiness ~ stress
Hp2	Positive emotional valence	Happiness ~ relax	Happiness > stress
Hp3	High life satisfaction	Happiness > relax	Happiness > stress

**Table 2:** A priori analysis to compute the required sample size in a repeated measures ANOVA.

Input		Output	
Effect size *f*	0.25	Noncentrality parameter *λ*	10.5
*α* err prob	0.05	Critical *F*	3.168246
Power (1 − *β* err prob)	0.8	Numerator df	2
Number of groups	1	Denominator df	54
Number of measurements	3	Total sample size	28
Corr among rep measures	0.5	Actual power	0.8124546
Nonsphericity correction *ε*	1		

**Table 3:** Hypotheses per each measure (− is for lower and + is for higher).

Dimension	Measure	Biomedical signal	Session type			Pairwise comparisons (Bonferroni correction)	
			Relax	Happiness	Stress	Happiness versus Relax	Happiness versus Stress
Higher arousal	HR	ECG	−	+	+	Sig.	−
	SC	GSR	−	+	+	Sig.	−
	Beta indexes	EEG	−	+	+	Sig.	−
	Pupil size	ET	−	+	+	Sig.	−
	Respiration depth	RSP	+	−	−	Sig.	−

Positive valence	EMG zygomatic	EMG	+	+	−	−	Sig.
	EMG Corrugator	EMC	−	−	+	−	Sig.
	Startle reflex	EMG	−	−	+	−	Sig.
	EEG Alpha Asymmetry	EEG	+	+	−	−	Sig.

Higher satisfaction	LF/HF	ECG	−	−	+	Sig.	Sig.
	NN50	ECG	+	+	−	Sig.	Sig.
	RMSSD	ECG	+	+	−	Sig.	Sig.
	HF power	ECG	+	+	−	Sig.	Sig.
	LF power	ECG	−	−	+	Sig.	Sig.

## References

[1] Congress, U.S.C. A declaration by the representatives of the United States of America.

[2] Russell JA (1979). Affective space is bipolar. *Journal of Personality and Social Psychology*.

[3] Lang PJ (1995). The emotion probe: studies of motivation and attention. *The American Psychologist*.

[4] Morris JD (1995). Observations: SAM: the self-assessment manikin—an efficient cross-cultural measurement of emotional response. *Journal of Advertising Research*.

[5] Rubin RB, Rubin AM, Graham E, Perse EM, Seibold D (2009). Self-Assessment Manikin. *Communication Research Measures II: A Sourcebook*.

[6] Backs RW, Da Silva SP, Han K (2005). A comparison of younger and older adults’ self-assessment Manikin ratings of affective pictures. *Experimental Aging Research*.

[7] Cipresso P, Serino S, Villani D (2012). Is your phone so smart to affect your state? An exploratory study based on psychophysiological measures. *Neurocomputing*.

[8] Bradley MM, Lang PJ (2000). Affective reactions to acoustic stimuli. *Psychophysiology*.

[9] Keil A, Bradley MM, Hauk O, Rockstroh B, Elbert T, Lang PJ (2002). Large-scale neural correlates of affective picture processing. *Psychophysiology*.

[10] Grühn D, Scheibe S (2008). Age-related differences in valence and arousal ratings of pictures from the international affective picture system (LAPS): do ratings become more extreme with age?. *Behavior Research Methods*.

[11] Kuhr B, Jacobi J, Krause C (2011). Arousal, valence, dominance...and desire? Evidence from an Erp study concerning the necessity of a new motivational dimension to describe affective states. *Psychophysiology*.

[12] Rozenkrants B, Polich J (2008). Affective ERP processing in a visual oddball task: arousal, valence, and gender. *Clinical Neurophysiology*.

[13] Jatupaiboon N, Pan-ngum S, Israsena P (2013). Real-time EEG-based happiness detection system. *The Scientific World Journal*.

[14] Rainville P, Bechara A, Naqvi N, Damasio AR (2006). Basic emotions are associated with distinct patterns of cardiorespiratory activity. *International Journal of Psychophysiology*.

[15] Cacioppo JT, Tassinary LG, Berntson GG (2007). *Handbook of Psychophysiology*.

[16] Mauri M, Cipresso P, Balgera A, Villamira M, Riva G (2011). Why is Facebook so successful? Psychophysiological measures describe a core flow state while using Facebook. *Cyberpsychology, Behavior, and Social Networking*.

[17] Magagnin V, Mauri M, Cipresso P (2010). Heart rate variability and respiratory sinus arrhythmia assessment of affective states by bivariate autoregressive spectral analysis. *Computers in Cardiology*.

[18] Mauri M, Magagnin V, Cipresso P Psychophysiological signals associated with affective states.

[19] Argyle M (2001). *The Psychology of Happiness*.

[20] David SA, Boniwell I, Ayers AC (2013). *The Oxford Handbook of Happiness*.

[21] Achor S (2010). *The Happiness Advantage: The Seven Principles of Positive Psychology That Fuel Success and Performance at Work*.

[22] Waterman AS (1993). Two conceptions of happiness: contrasts of personal expressiveness (Eudaimonia) and hedonic enjoyment. *Journal of Personality and Social Psychology*.

[23] Walker CO, Winn TD, Lutjens RM (2012). Examining relationships between academic and social achievement goals and routes to happiness. *Education Research International*.

[24] Headey B, Kelley J, Wearing A (1993). Dimensions of mental health: life satisfaction, positive affect, anxiety and depression. *Social Indicators Research*.

[25] Steca P, Greco A, Monzani D (2013). How does illness severity influence depression, health satisfaction and life satisfaction in patients with cardiovascular disease? The mediating role of illness perception and self-efficacy beliefs. *Psychology and Health*.

[26] Nes RB, Czajkowski NO, Røysamb E, Orstavik RE, Tambs K, Reichborn-Kjennerud T (2013). Major depression and life satisfaction: a population-based twin study. *Journal of Affective Disorders*.

[27] Gnilka PB, Ashby JS, Noble CM (2013). Adaptive and maladaptive perfectionism as mediators of adult attachment styles and depression, hopelessness, and life satisfaction. *Journal of Counseling and Development*.

[28] Eskin M, Akyol A, Çelik EY, Gültekin BK (2013). Social problem-solving, perceived stress, depression and life-satisfaction in patients suffering from tension type and migraine headaches. *Scandinavian Journal of Psychology*.

[29] Yorgason J, Choi H, Gustafson K, Godfrey W, Bond A (2012). Daily associations between family and community support with daily levels of depression, anxiety, and life satisfaction. *Gerontologist*.

[30] Greco A, Monzani D, Pancani L, Cappelletti ER, D'Addario M, Steca P (2012). Relationships of illness severity with depression, health- and life-satisfaction in patients with cardiovascular diseases. *Psychology and Health*.

[31] Garver K, Bogda K, Westrup R (2012). Effects of resistance training on mood, life satisfaction, and depression. *Gerontologist*.

[32] Fagerström C, Lindwall M, Berg AI, Rennemark M (2012). Factorial validity and invariance of the life satisfaction Index in older people across groups and time: addressing the heterogeneity of age, functional ability, and depression. *Archives of Gerontology and Geriatrics*.

[33] Britton PC, Ouimette PC, Bossarte RM (2012). The effect of depression on the association between military service and life satisfaction. *Quality of Life Research*.

[34] Bamishigbin ON, Carver C, Spillers R (2012). Effects of optimism, benefit-finding, and spirituality on depression and life satisfaction in cancer caregivers. *Annals of Behavioral Medicine*.

[35] Yamasaki K, Sasaki M, Uchida K, Katsuma L (2011). P02-109—effects of positive and negative affect and emotional supression on short-term life satisfaction and depression: considering activation of affect. *European Psychiatry*.

[36] Grinde B (2009). An evolutionary perspective on the importance of community relations for quality of life. *TheScientificWorldJOURNAL*.

[37] Carney RM, Rich MW, TeVelde A, Saini J, Clark K, Freedland KE (1988). The relationship between heart rate, heart rate variability and depression in patients with coronary artery disease. *Journal of Psychosomatic Research*.

[38] Yeragani VK, Pohl R, Balon R (1991). Heart rate variability in patients with major depression. *Psychiatry Research*.

[39] Yeragani K, Berger R, Pohl R (1993). Heart-rate-variability in patients with panic disorder and depression. *Biological Psychiatry*.

[40] Runions J, Kamath MV, Fallen EL (1994). Heart-rate-variability and depression—a linkage. *Circulation*.

[41] Yeragani VK, Balon R, Pohl R, Ramesh C (1995). Depression and heart rate variability. *Biological Psychiatry*.

[42] Choi C, Kim K, Kim C, Kim SH, Choi W (2011). Reactivity of heart rate variability after exposure to colored lights in healthy adults with symptoms of anxiety and depression. *International Journal of Psychophysiology*.

[43] Song BA, Yoo S, Kang H (2011). Post-traumatic stress disorder, depression, and heart-rate variability among North Korean defectors. *Psychiatry Investigation*.

[44] Tonhajzerova I, Ondrejka I, Turianikova Z (2011). P01-359—heart rate variability in adolescent major depression. *European Psychiatry*.

[45] Celik A, Ozturk A, Ozbek K, Ceyhan K, Kadi H, Koc F (2012). The value of heart rate variability and turbulence for discriminating the true coronary artery disease from false positive results in patients with ST segment depression without angina during exercise stress testing. *Circulation*.

[46] Kemp AH (2012). Reply to: are antidepressants good for the soul but bad for the matter? Using noninvasive brain stimulation to detangle depression/antidepressants effects on heart rate variability and cardiovascular risk. *Biological Psychiatry*.

[47] Kemp AH, Quintana DS, Felmingham KL, Matthews S, Jelinek HF (2012). Depression, comorbid anxiety disorders, and heart rate variability in physically healthy, unmedicated patients: implications for cardiovascular risk. *PLoS ONE*.

[48] Mennin DS, Fresco DM, Aldao A (2012). Phasic heart rate variability changes predict clinical outcomes of emotion regulation therapy for generalized anxiety disorder and comorbid depression. *Psychophysiology*.

[49] Munk PS, Isaksen K, Brønnick K, Kurz MW, Butt N, Larsen AI (2012). Symptoms of anxiety and depression after percutaneous coronary intervention are associated with decreased heart rate variability, impaired endothelial function and increased inflammation. *International Journal of Cardiology*.

[50] Benvenuti SM, Patron E, Favretto G, Gasparotto R, Palomba D (2013). Depression and reduced heart rate variability after cardiac surgery: the mediating role of emotion regulation. *Psychophysiology*.

[51] Harte CB, Liverant GI, Sloan DM (2013). Association between smoking and heart rate variability among individuals with depression. *Annals of Behavioral Medicine*.

[52] Huang HT, Wan KS (2013). Heart rate variability in junior high school students with depression and anxiety in Taiwan. *Acta Neuropsychiatrica*.

[53] Suh S, Ellis RJ, Sollers 3rd JJ, Thayer JF, Yang HC, Emery CF (2013). The effect of anxiety on heart rate variability, depression, and sleep in chronic obstructive pulmonary disease. *Journal of Psychosomatic Research*.

[54] Lang PJ, Bradley MM, Cuthbert BN (2008). International affective picture system (IAPS): affective ratings of pictures and instruction manual.

[55] Bradley MM, Lang PJ (1999). International affective digitized sounds (IADS): stimuli, instruction manual and affective ratings.

[56] Bradley MM, Lang PJ (1999). Affective norms for English words (ANEW): stimuli, instruction manual and affective ratings.

[57] Bradley MM, Lang PJ (2007). Affective norms for English text (ANET): affective ratings of text and instruction manual.

[58] Grühn D, Smith J (2008). Characteristics for 200 words rated by young and older adults: age-dependent evaluations of German adjectives (AGE). *Behavior Research Methods*.

[59] Leite J, Carvalho S, Galdo-Alvarez S, Alves J, Sampaio A, Gonçalves ÓF (2012). Affective picture modulation: valence, arousal, attention allocation and motivational significance. *International Journal of Psychophysiology*.

[60] von Leupoldt A, Taube K, Henkhus M, Dahme B, Magnussen H (2010). The impact of affective states on the perception of dyspnea in patients with chronic obstructive pulmonary disease. *Biological Psychology*.

[61] Von Leupoldt A, Hartle-Bremerich B, Dahme B (2005). Application of video glasses for sustained affective picture presentations: a comparison with video projector presentations. *Behavior Research Methods*.

[62] Csikszentmihalyi M (1975). *Beyond Boredom and Anxiety*.

[63] Csikszentmihalyi M (1990). *Flow: The Psychology of Optimal Experience*.

[64] Riva G, Baños RM, Botella C, Wiederhold BK, Gaggioli A (2012). Positive technology: using interactive technologies to promote positive functioning. *Cyberpsychology, Behavior, and Social Networking*.

[65] Wiederhold BK, Riva G (2012). Positive technology supports shift to preventive, integrative health. *Cyberpsychology, Behavior, and Social Networking*.

[66] Serino S, Cipresso P, Gaggioli A, Riva G (2013). The potential of pervasive sensors and computing for positive technology: the interreality paradigm. *Pervasive and Mobile Sensing and Computing for Healthcare*.

[67] Villani D, Grassi A, Cognetta C, Toniolo D, Cipresso P, Riva G (2013). Self-help stress management training through mobile phones: an experience with oncology nurses. *Psychological Services*.

[68] Cipresso P, Gaggioli A, Serino S (2012). EEG alpha asymmetry in virtual environments for the assessment of stress-related disorders. *Studies in Health Technology and Informatics*.

[69] Camm AJ, Malik M, Bigger JT (1996). Heart rate variability: standards of measurement, physiological interpretation, and clinical use. *Circulation*.

[70] Society for Psychophysiological Research Ad Hoc Committee on Electrodermal Measures (2012). Publication recommendations for electrodermal measurements. *Psychophysiology*.

[71] Fowles DC, Christie MJ, Edelberg R (1981). Publication recommendations for electrodermal measurements. *Psychophysiology*.

[72] Larsen JT, Norris CJ, Cacioppo JT (2003). Effects of positive and negative affect on electromyographic activity over zygomaticus major and corrugator supercilii. *Psychophysiology*.

[73] Veldhuizen IJT, Gaillard AWK (2001). Can differences in fatigue be reflected by differences in tonic EMG corrugator supercilii muscle activity?. *Psychophysiology*.

[74] Goodmurphy CW, Ovalle WK (1999). Morphological study of two human facial muscles: orbicularis oculi and corrugator supercilii. *Clinical Anatomy*.

[75] Blumenthal TD, Cuthbert BN, Filion DL, Hackley S, Lipp OV, van Boxtel A (2005). Committee report: guidelines for human startle eyeblink electromyographic studies. *Psychophysiology*.

[76] Kunzmann U, Kupperbusch CS, Levenson RW (2005). Behavioral inhibition and amplification during emotional arousal: a comparison of two age groups. *Psychology and Aging*.

[77] Bagić AI, Knowlton RC, Rose DF, Ebersole JS (2011). American clinical magnetoencephalography society clinical practice guideline 3: MEG-EEG reporting. *Journal of Clinical Neurophysiology*.

[78] Bagić AI, Knowlton RC, Rose DF, Ebersole JS (2011). American clinical magnetoencephalography society clinical practice guideline 1: recording and analysis of spontaneous cerebral activity. *Journal of Clinical Neurophysiology*.

[79] Nikulin VV, Brismar T (2004). Long-range temporal correlations in alpha and beta oscillations: effect of arousal level and test-retest reliability. *Clinical Neurophysiology*.

[80] Debener S, Beauducel A, Fiehler K, Rabe S, Brocke B (2001). Frontal EEG alpha asymmetry and affective style: are individual differences related to fundamental dimensions of emotion?. *Psychophysiology*.

[81] Gordon E, Palmer DM, Cooper N (2010). EEG alpha asymmetry in schizophrenia, depression, PTSD, panic disorder, ADHD and conduct disorder. *Clinical EEG and Neuroscience*.

[82] Mathersul D, Williams LM, Hopkinson PJ, Kemp AH (2008). Investigating models of affect: relationships among EEG alpha asymmetry, depression, and anxiety. *Emotion*.

[83] de Raedt R, Franck E, Fannes K, Verstraeten E (2008). Is the relationship between frontal EEG alpha asymmetry and depression mediated by implicit or explicit self-esteem?. *Biological Psychology*.

[84] Nusslock R, Shackman AJ, McMenamin BW, Greischar LL, Kovacs M, Davidson RJ (2008). Anxiety moderates relations between frontal EEG alpha asymmetry and depression. *Psychophysiology*.

[85] Nusslock R, Shackman AJ, Greischar LL, McMenamin BW, Kovacs M, Davidson RJ (2007). Frontal EEG alpha asymmetry in depression: the role of clinical state and emotion regulation. *Psychophysiology*.

[86] Tops M, Wijers AA, van Staveren ASJ (2005). Acute cortisol administration modulates EEG alpha asymmetry in volunteers: relevance to depression. *Biological Psychology*.

[87] Niemiec AJ, Lithgow BJ Alpha-band characteristics in EEG spectrum indicate reliability of frontal brain asymmetry measures in diagnosis of depression.

[88] Diego MA, Field T, Hernandez-Reif M (2001). CES-D depression scores are correlated with frontal EEG alpha asymmetry. *Depression and Anxiety*.

[89] Debener S, Beauducel A, Nessler D, Brocke B, Heilemann H, Kayser J (2000). Is resting anterior EEG alpha asymmetry a trait marker for depression? Findings for healthy adults and clinically depressed patients. *Neuropsychobiology*.

[90] Urry HL, Hitt SK, Allen JJB (1999). Internal consistency and test-retest stability of resting EEG alpha asymmetry in major depression. *Psychophysiology*.

[91] Gotlib IH, Ranganath C, Rosenfeld JP (1998). Frontal EEG alpha asymmetry, depression, and cognitive functioning. *Cognition and Emotion*.

[92] Lykins AD, Meana M, Kambe G (2006). Detection of differential viewing patterns to erotic and non-erotic stimuli using eye-tracking methodology. *Archives of Sexual Behavior*.

[93] Cipresso P, Mauri M, Balgera A, Romanò E, Villamira M (2010). Synchronization of a biofeedback system with an eye tracker through an audiovisual stimulus marker. *Applied Psychophysiology and Biofeedback*.

[94] Cipresso P (2011). Synchronizing physiological signals acquired from biofeedback equipment and eye-tracker systems. *Applied Psychophysiology and Biofeedback*.

[95] Oliveira JM, Volchan E, Vargas CD, Gleiser S, David IA (2012). Box for interaction with objects (BIO): a new device to synchronize the presentation of objects with electrophysiological recordings. *Behavior Research Methods*.

[96] Emam KE, Moreau K, Jonker E (2011). How strong are passwords used to protect personal health information in clinical trials?. *Journal of Medical Internet Research*.

[97] Cipresso P, Gaggioli A, Serino S, Cipresso S, Riva G (2012). How to create memorizable and strong passwords. *Journal of Medical Internet Research*.

[98] Brumen B, Heričko M, Rozman I, Hölbl M (2013). Security Analysis and Improvements to the PsychoPass Method. *Journal of Medical Internet Research*.

[99] Lee K, Gilmore DF (2006). Statistical experimental design for bioprocess modeling and optimization analysis: repeated-measures method for dynamic biotechnology process. *Applied Biochemistry and Biotechnology*.

[100] Bakker M, Wicherts JM (2011). The (mis)reporting of statistical results in psychology journals. *Behavior Research Methods*.

[101] Kuehl RO (2000). *Design of Experiments: Statistical Principles of Research Design and Analysis*.

[102] Oehlert GW (2000). *A First Course in Design and Analysis of Experiments*.

[103] Girden ER (1992). *ANOVA: Repeated Measures*.

[104] Faul F, Erdfelder E, Lang A, Buchner A (2007). G*Power 3: a flexible statistical power analysis program for the social, behavioral, and biomedical sciences. *Behavior Research Methods*.

[105] Sleight P, La Rovere MT, Mortara A (1995). Physiology and pathophysiology of heart rate and blood pressure variability in humans: is power spectral analysis largely an index of baroreflex gain? (Clinical Science (1995) 88 (103–109)). *Clinical Science*.

[106] Picard RW (1997). *Affective Computing*.

[107] Shiffman S, Stone AA (1998). Ecological momentary assessment: a new tool for behavioral medicine research. *Technology and Methods in Behavioral Medicine*.

[108] Shiffman S, Stone AA, Hufford MR (2008). Ecological momentary assessment. *Annual Review of Clinical Psychology*.

[109] Dockray S, Grant N, Stone AA, Kahneman D, Wardle J, Steptoe A (2010). A comparison of affect ratings obtained with ecological momentary assessment and the day reconstruction method. *Social Indicators Research*.

